# Mammary and gut microbiome profiles in experimentally induced murine mastitis

**DOI:** 10.1128/mra.01428-25

**Published:** 2026-01-27

**Authors:** Md. Morshedur Rahman, Md Abu Ahsan Gilman, M. Nazmul Hoque

**Affiliations:** 1 Molecular Biology and Bioinformatics Laboratory, Department of Gynecology, Obstetrics and Reproductive Health, Gazipur Agricultural University 198780, Gazipur, Bangladesh; Nanchang University, Nanchang, Jiangxi, China

**Keywords:** mastitis, mouse model, amplicon sequencing, mammary and gut microbiome

## Abstract

*16S rRNA* amplicon sequencing of mammary and gut samples (*N* = 32) from healthy (*n* = 3) and experimentally induced mastitis mice (*n* = 13) revealed *Lactobacillus*-dominated bacteriomes, low archaeal diversity, and significant mastitis-associated shifts. These results provide a foundational data set for understanding mastitis-causing major pathogen-driven dysbiosis, host-microbiome interactions, and disease-specific microbial community dynamics.

## ANNOUNCEMENT

Mastitis is an inflammatory condition of mammary glandular tissue that causes pathological changes in udder and alters local immune responses (1–3). Experimental mouse models are widely used to study host-pathogen interactions, disease progression, and the associated microbiome dysbiosis (4, 5). Mastitis induction disrupts mammary and gut microbial communities, reflecting systemic inflammatory effects that shape disease severity, tissue damage, and recovery dynamics.

Sixteen pregnant Swiss albino mice (procured from icddr,b) were used in this study. Mastitis was experimentally induced in 13 mice by oral administration of *Staphylococcus aureus*, *Klebsiella pneumoniae*, and *Escherichia coli* (individually or in combination, 100 µL at 10⁶ CFU/mL) at Gazipur Agricultural University (24.03°N, 90.39°E). Three mice served as healthy controls. A total of 32 samples, comprising 16 mammary tissue (MT) and 16 fecal samples (FSs), were collected following a previously established protocol (6). MT was aseptically collected after euthanasia, washed with sterile PBS, and stored at −20°C. Fecal pellets (80 mg) were aseptically collected, resuspended in sterile PBS, vortexed, centrifuged to remove debris, and processed in lysis buffer. DNA was extracted from 50 mg MT and 400 µL FS suspensions using the Maxwell 16 Tissue DNA and Maxwell 16 FFS Nucleic Acid Extraction Kits (Promega, USA), following the manufacturer’s instructions. PCR negative controls were included to monitor contamination. Amplicon libraries were prepared using the Nextera XT Index Kit (Illumina, USA), and the V3–V4 regions were amplified using 341F and 806R primers (7, 8) with a 30-cycle PCR protocol, under conditions of 98°C denaturation, 50°C annealing, and 72°C extension, followed by a final extension at 72°C (9, 10). Sequencing was conducted on the Illumina MiSeq platform using the MiSeq Reagent Kit v3 (2 × 300 bp paired-end). Reads were quality filtered using fastp v.1.0.1 (Q20, min length 50 bp) (11) and clustered into operational taxonomic units (OTUs) at 97% sequence similarity using a *de novo* approach in the CZ ID pipeline v.7.1 (12). Taxonomic classification was performed in CZ ID using alignment-based methods against the NCBI nucleotide and non-redundant protein reference databases. Phyloseq v1.42 implemented in R v4.4.0 (13) was subsequently used for downstream data curation and microbiome analyses. Default parameters were used except where otherwise specified.

From 32 samples, 70,541,932 raw reads were obtained, averaging 2,204,435/sample. After quality filtering, 44,007,193 reads (62.38%) were assigned to 102 OTUs (Table 1) representing 11 bacterial phyla, 18 classes, 39 orders, 65 families, and 98 genera. Archaeal diversity was low, consisting of a single phylum with two classes, two orders, two families, and three genera. OTU richness ranged from 40 (G1M1U1) to 53 (G3M3U3) in MT samples and 45 (G3M1F1) to 56 (G1M1F1 and G4M4F4) in FS samples (Table 1). Across all samples, *Lactobacillus* (52.12%) dominated the bacteriome community, followed by *Azospirillum* (8.79%), *Streptomyces* (7.98%), *Corynebacterium* (4.53%), *Bifidobacterium* (3.49%), and *Streptococcus* (3.47%). The remaining bacterial genera exhibited relatively lower abundances (<3.4%). Archaea were detected at very low levels, represented by *Methanohalophilus* (0.018%), *Thermococcus* (0.006%), and *Pyrococcus* (0.003%). This amplicon sequencing-based profiling of mammary and gut microbiomes from healthy and mastitis-induced mice provides a foundation for studying mastitis-associated microbial shifts and host-microbiome interactions.

**TABLE 1:** Sequencing summary and taxonomic classification for murine mastitis samples^*a*^^,^^*b*^

Sample ID	Location	Coordinate	Source	Groups	Condition	No. of raw reads	No. of quality reads	No. of mapped reads	No. of observed OTUs	SRA accession
G1M1F1	GAU	24.0360 N, 90.3963 E	FS	KPN	Mastitis	1,416,964	1,188,050	1,187,988	56	SRR36172640
G1M1U1	GAU	24.0360 N, 90.3963 E	MT	KPN	Mastitis	1,321,894	706,204	704,981	40	SRR36172639
G1M2F2	GAU	24.0360 N, 90.3963 E	FS	KPN	Mastitis	1,330,728	1,184,504	1,184,420	49	SRR36172628
G1M2U2	GAU	24.0360 N, 90.3963 E	MT	KPN	Mastitis	2,924,914	1,476,586	1,474,642	51	SRR36172617
G1M3F3	GAU	24.0360 N, 90.3963 E	FS	KPN	Mastitis	1,983,774	1,447,952	1,447,897	48	SRR36172614
G1M3U3	GAU	24.0360 N, 90.3963 E	MT	KPN	Mastitis	2,721,526	1,615,712	1,615,278	42	SRR36172613
G2M1F1	GAU	24.0360 N, 90.3963 E	FS	SA	Mastitis	2,593,078	1,390,982	1,388,218	49	SRR36172612
G2M1U1	GAU	24.0360 N, 90.3963 E	MT	SA	Mastitis	2,693,618	1,454,542	1,451,844	51	SRR36172611
G2M2F2	GAU	24.0360 N, 90.3963 E	FS	SA	Mastitis	1,845,760	1,413,478	1,413,269	50	SRR36172610
G2M2U2	GAU	24.0360 N, 90.3963 E	MT	SA	Mastitis	2,727,092	1,373,932	1,371,204	41	SRR36172609
G2M3F3	GAU	24.0360 N, 90.3963 E	FS	SA	Mastitis	1,565,578	1,337,964	1,337,882	47	SRR36172638
G2M3U3	GAU	24.0360 N, 90.3963 E	MT	SA	Mastitis	2,407,386	1,360,938	1,359,717	47	SRR36172637
G3M1F1	GAU	24.0360 N, 90.3963 E	FS	EC	Mastitis	1,092,858	952,678	952,616	45	SRR36172636
G3M1U1	GAU	24.0360 N, 90.3963 E	MT	EC	Mastitis	2,871,366	1,616,822	1,614,954	51	SRR36172635
G3M2F2	GAU	24.0360 N, 90.3963 E	FS	EC	Mastitis	2,116,842	1,489,602	1,489,526	52	SRR36172634
G3M2U2	GAU	24.0360 N, 90.3963 E	MT	EC	Mastitis	2,884,544	1,598,262	1,595,254	46	SRR36172633
G3M3F3	GAU	24.0360 N, 90.3963 E	FS	EC	Mastitis	2,046,790	1,683,532	1,683,402	47	SRR36172632
G3M3U3	GAU	24.0360 N, 90.3963 E	MT	EC	Mastitis	2,280,466	1,202,184	1,197,218	53	SRR36172631
G4M1F1	GAU	24.0360 N, 90.3963 E	FS	Combined	Mastitis	2,269,266	1,649,948	1,649,861	47	SRR36172630
G4M1U1	GAU	24.0360 N, 90.3963 E	MT	Combined	Mastitis	2,635,936	1,261,410	1,258,818	44	SRR36172629
G4M2F2	GAU	24.0360 N, 90.3963 E	FS	Combined	Mastitis	1,647,952	1,365,860	1,365,633	53	SRR36172627
G4M2U2	GAU	24.0360 N, 90.3963 E	MT	Combined	Mastitis	2,620,506	1,312,838	1,310,891	45	SRR36172626
G4M3F3	GAU	24.0360 N, 90.3963 E	FS	Combined	Mastitis	1,539,286	1,337,306	1,337,256	45	SRR36172625
G4M3U3	GAU	24.0360 N, 90.3963 E	MT	Combined	Mastitis	2,021,180	1,119,806	1,117,650	44	SRR36172624
G4M4F4	GAU	24.0360 N, 90.3963 E	FS	Combined	Mastitis	1,607,924	1,226,428	1,226,186	56	SRR36172623
G4M4U4	GAU	24.0360 N, 90.3963 E	MT	Combined	Mastitis	2,430,094	1,306,342	1,303,885	48	SRR36172622
G5M1F1	GAU	24.0360 N, 90.3963 E	FS	Control	Healthy	2,754,466	1,502,200	1,500,775	52	SRR36172621
G5M1U1	GAU	24.0360 N, 90.3963 E	MT	Control	Healthy	2,976,310	1,640,464	1,638,360	50	SRR36172620
G5M2F2	GAU	24.0360 N, 90.3963 E	FS	Control	Healthy	1,911,170	1,413,546	1,413,314	48	SRR36172619
G5M2U2	GAU	24.0360 N, 90.3963 E	MT	Control	Healthy	2,859,526	1,515,394	1,514,604	51	SRR36172618
G5M3F3	GAU	24.0360 N, 90.3963 E	FS	Control	Healthy	1,859,684	1,503,474	1,503,286	48	SRR36172616
G5M3U3	GAU	24.0360 N, 90.3963 E	MT	Control	Healthy	2,598,366	1,398,438	1,396,364	47	SRR36172615

^*a*^ Includes sample metadata, read processing statistics (raw, quality-filtered, mapped), and the assignment of 102 OTUs from MT and (FSs with corresponding SRA accession numbers for 16S rRNA sequences.

^*b*^ GAU: Gazipur Agricultural University (24.03° N, 90.39° E), KPN, *K. pneumoniae*; SA, *S. aureus*; EC: *E. coli*; and control: healthy mice.

## Data Availability

The 16S rRNA amplicon sequencing data are available at the NCBI Sequence Read Archive (SRA) under BioProject accession number PRJNA1368461. The accession numbers for all 32 SRA experiments are listed in Table 1.
